# Calving rates at tidewater glaciers vary strongly with ocean temperature

**DOI:** 10.1038/ncomms9566

**Published:** 2015-10-09

**Authors:** Adrian Luckman, Douglas I. Benn, Finlo Cottier, Suzanne Bevan, Frank Nilsen, Mark Inall

**Affiliations:** 1 Department of Geography, College of Science, Swansea University, SA2 8PP, UK; 2 Department of Arctic Geophysics, University Centre in Svalbard, Po Box 156, 9171 Longyearbyen, Norway; 3 Department of Arctic Geology, University Centre in Svalbard, Po Box 156, 9171 Longyearbyen, Norway; 4 Department of Geography, University of St Andrews, St Andrews, Fife, KY16 9AJ, UK; 5 Scottish Association for Marine Science, Oban PA37 1QA, UK; 6 Faculty of Biosciences, Fisheries and Economics, UiT The Arctic University of Norway, Po Box 6050 Langnes 9037 Tromsø, Norway; 7 The Geophysical Institute, University of Postboks 7803 5020 Bergen, Norway

## Abstract

Rates of ice mass loss at the calving margins of tidewater glaciers (frontal ablation rates) are a key uncertainty in sea level rise projections. Measurements are difficult because mass lost is replaced by ice flow at variable rates, and frontal ablation incorporates sub-aerial calving, and submarine melt and calving. Here we derive frontal ablation rates for three dynamically contrasting glaciers in Svalbard from an unusually dense series of satellite images. We combine ocean data, ice-front position and terminus velocity to investigate controls on frontal ablation. We find that frontal ablation is not dependent on ice dynamics, nor reduced by glacier surface freeze-up, but varies strongly with sub-surface water temperature. We conclude that calving proceeds by melt undercutting and ice-front collapse, a process that may dominate frontal ablation where submarine melt can outpace ice flow. Our findings illustrate the potential for deriving simple models of tidewater glacier response to oceanographic forcing.

## Abstract (web summary)

Tidewater glacier calving is assumed to be dependent on ice dynamics, but direct evidence is lacking. Here, Luckman *et al*. use satellite data to derive frontal ablation rates for dynamically contrasting Svalbard glaciers, and show that frontal ablation rate varies primarily with sub-surface ocean temperature.

## Graphical abstract

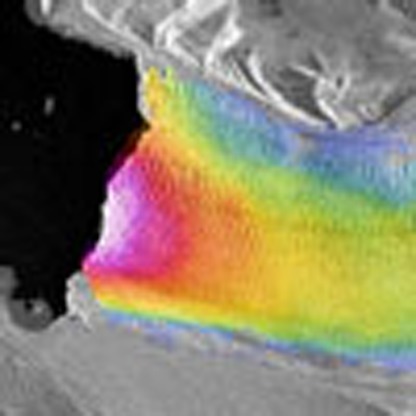

Loss of mass at the termini of tidewater glaciers, or frontal ablation, occurs by a combination of calving and submarine melting^1^,^2^. In addition to its direct contribution, submarine melting can amplify calving by undercutting and destabilizing the glacier front^3^. Several recent studies have concluded that submarine melting can dominate mass loss at tidewater termini, particularly where glaciers are impacted by incursions of warm water from the continental shelf or beyond^2^,^4^,^5^. However, there remains a distinct lack of direct measurements of either calving or subaqueous melt rates. Almost all estimates of the latter are based on calculations of the frontal heat budget using oceanographic data, for example, refs 1, 6, 7, and because these estimates rely on extrapolations from spatially and temporally limited CTD and current measurements, they tend to be subject to large uncertainties. Some attempt has been made to directly measure frontal ablation rates at tidewater glaciers^8^,^9^ but temporal and spatial resolution is often poor, and winter months are inadequately sampled. This scarcity of direct measurements of frontal ablation rates and their relationship to conditions both above and below the waterline places severe limits on our ability to predict the response of tidewater glaciers to oceanographic forcing^10^.

We address this problem by combining high temporal and spatial resolution satellite radar data with oceanographic and meteorological time series to investigate controls on frontal ablation rates. We derive time series of frontal velocities, terminus positions and frontal ablation rates for three Svalbard tidewater glaciers using 11-day repeat, 2 m resolution, TerraSAR-X images spanning more than a year. Analysis of these data allows us to quantify evolution of frontal ablation rates in unprecedented detail through a complete seasonal cycle, and to identify the key environmental controls on calving behaviour. We find that rates of frontal ablation are not dependent on glacier dynamics, nor reduced by the onset of glacier surface freeze-up, but closely follow the temperature of the fjord waters at depth. Our findings imply that submarine melt undercut and collapse is the dominant calving mechanism at these glaciers, and that frontal ablation is therefore controlled primarily by ocean temperatures.

## Results

### Glaciers studied

**Figure 1:**
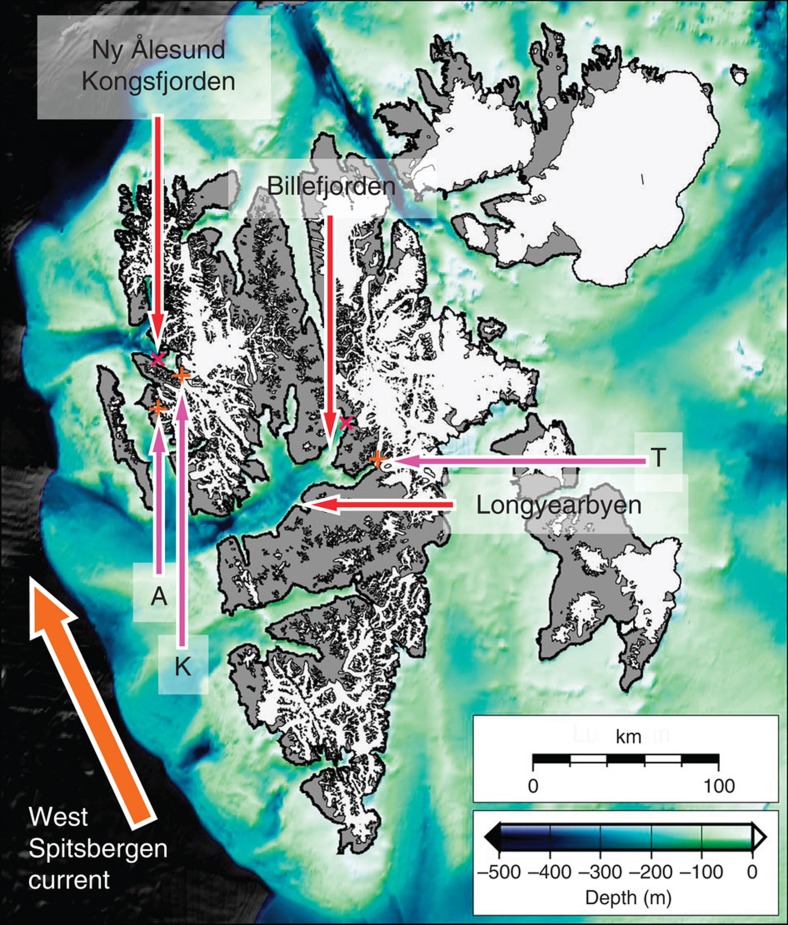
Svalbard location map. Arrows show positions of glaciers (K=Kronebreen, T=Tunabreen, A=Aavatsmarkbreen), weather stations, and moorings. Background bathymetry from IBCAO Version 3.0 (ref. 35) illustrates the possible routes for warm water from the WSC via troughs to fjords.

We choose three glaciers for their contrasting dynamic characteristics (Fig. 1): Kronebreen, a glacier with one of the highest flux rates in Svalbard; Tunabreen, a quiescent-phase surge-type glacier; and Aavatsmarkbreen, an active-phase surge-type glacier that began a surge during our observation period. In addition to their contrasting dynamical characteristics, these glaciers also vary in their fjord settings (Fig. 1): Kronebreen terminates in Kongsfjorden which has a deep, unrestricted connection to the warm West-Spitsbergen Current (WSC), but has a relatively shallow connection between the outer and inner parts of the fjord at ∼60 m depth. Tunabreen terminates in Tempelfjorden which is shallower and further removed from the WSC than Kongsfjorden (Fig. 1). Aavatsmarkbreen terminates in Forlandsundet, which is as close to the WSC as Kronebreen, but lacks a direct, deep connection.

### Calving rate measurement and potential controls

The rate of ice loss from a tidewater glacier terminus is the sum of that due to calving, and that due to submarine melting, and is commonly referred to as the frontal ablation rate 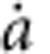 (ref. 2). A change in ice-front position over time must equal the rate at which ice is delivered to the terminus minus 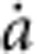. Thus satellite data of sufficient spatial resolution may be used to calculate this quantity through regular measurements of ice-front position, from which d*l*/d*t* may be derived, and of terminus ice speed *U*_*T*_:


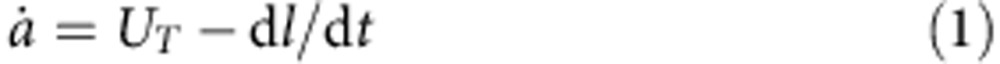


We use equation (1) to calculate the frontal ablation rate between pairs of TerraSAR-X images from surface velocities derived by feature tracking, and ice-front position change derived by manual digitization and geographical information systems (GIS) (Methods section). The images, in ‘Stripmap' mode (∼2 m ground range pixel size), were acquired every orbital cycle for 19 months during 2013 and early 2014, providing an image pair every 11 days barring a few acquisition failures. In the cases of Kronebreen and Aavatsmarkbreen, we incorporate images from more than one satellite track, giving a greater frequency of observations during some time periods.

To investigate the controls on measured frontal ablation rates we explore a variety of environmental variables that have previously been associated with calving behaviour. These include local weather data (air temperature and precipitation), sea-surface temperature (SST), fjord ice presence and water temperatures at depth in the glacier fjords (henceforth ‘sub-surface temperatures'; Methods section).

### Glacier dynamic behaviour

**Figure 2:**
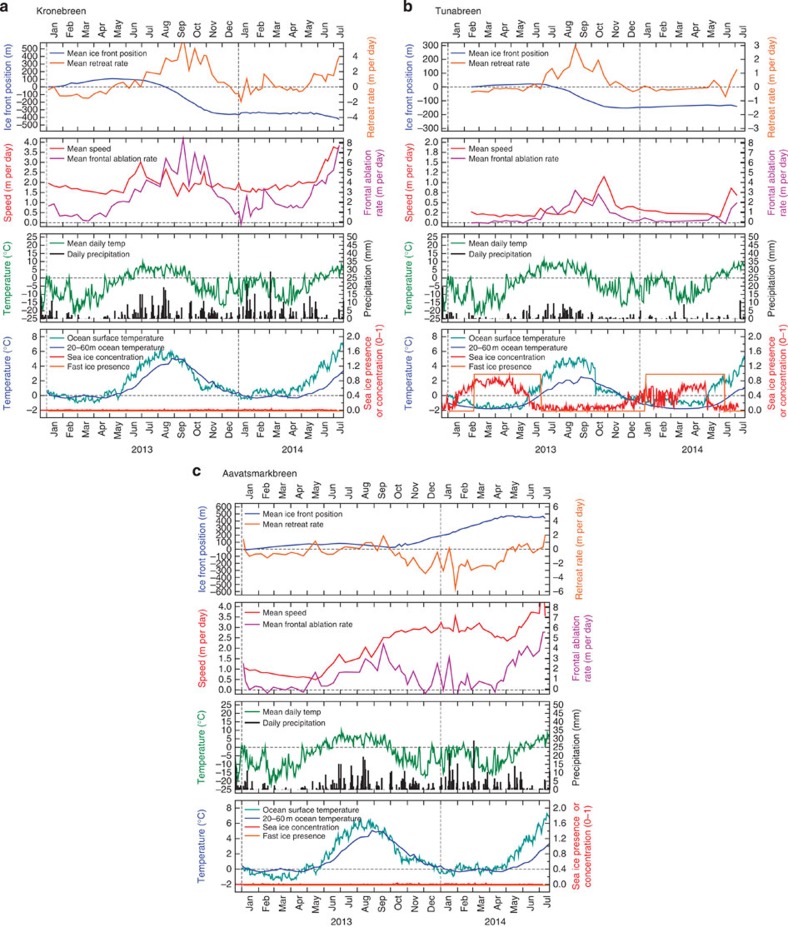
Glacier time series. Data derived from a series of 11-day repeat TerraSAR-X images from 2013 to 2014 for three contrasting Svalbard glaciers to illustrate the frontal ablation rate and its two key components: the rate of change of ice-front position and the terminus speed. Also shown are: temperature and precipitation from the nearest weather station (Ny Ålesund for (**a**) Kronebreen and (**c**) Aavatsmarkbreen; Longyearbyen for (**b**) Tunabreen); OSTIA SST and sea ice concentration^32^ from close to the ice front; fast ice presence in each fjord immediately in front of the ice front; and mean weekly water temperature between 20 and 60 m from the nearest ocean mooring. For (**a**,**c**) these data are from Kongsfjorden (Fig. 1); for (**b**) these data are from Billefjorden. Dashed vertical lines indicate year transitions.

Glacier terminus speeds (Fig. 2) show significantly different behaviours between the three glaciers during our observation period. Kronebreen has a winter speed of 1.5–2 m per day, with summer peaks of 3–4 m per day associated with positive air temperatures and periods of high rainfall. The winter speed of Tunabreen is only 0.2 m per day, rising to a peak of ∼1 m per day in October. Aavatsmarkbreen flowed at ∼1 m per day before its surge but by November 2013 had attained ∼3 m per day, a speed maintained without major fluctuations until May 2014 when a further summer speed-up occurred. These different behaviours reflect contrasting controls on the terminus dynamics of the three glaciers. The summer acceleration of Kronebreen is consistent with enhanced basal lubrication from surface melt water and precipitation^11^, and is in accord with reports of seasonal dynamic variation at tidewater glaciers elsewhere^8^,^9^,^12^,^13^. Detectable motion of the quiescent Tunabreen is confined to the frontal zone, where high longitudinal stress gradients induce outward stretching of the ice. Stretching rates are proportional to ice cliff height, which exhibits systematic seasonal variations in response to calving losses^14^. The sustained high velocities of Aavatsmarkbreen between summer 2013 and the end of our time series are characteristic of a surge, possibly modulated by the influx of surface melt water during the summer months.

Mean ice-front positions at Kronebreen and Tunabreen reveal comparable seasonal patterns, although of different magnitudes (Fig. 2). Both glaciers advance modestly during winter and retreat strongly during summer and autumn, a period associated with greater seismic activity^13^, with net annual retreats of ∼350 and ∼150 m, respectively. Similar patterns of seasonality in ice-front position are seen in Alaska^15^ and Greenland^16^. Aavatsmarkbreen followed the same pattern in spring 2013, but then advanced by ∼500 m between the start of the surge in summer 2013 and May 2014, after which it began to retreat.

By combining ice-front positions with terminus velocities (equation (1)) we quantify frontal ablation rates and their seasonal variation at an unprecedented temporal resolution (Fig. 2). Maximum frontal ablation rates for Kronebreen, Tunabreen and Aavatsmarkbreen are ∼8, ∼3 and ∼5 m per day, respectively. Despite their diverse dynamic behaviours and fjord settings, the magnitudes of these frontal ablation rates are notably comparable, and their seasonal patterns very similar. Rates for all three glaciers peak in September and October and continue at a high level well after air temperature has fallen consistently below 0 °C. Frontal ablation lags air and SSTs by 1–2 months but is in synchrony with the peak in sub-surface ocean temperature. We note that frontal ablation rates at a sample of Alaskan Glaciers appear to peak earlier in the summer^9^, but that a strongly similar seasonal pattern of calving rate to our glaciers is seen at Columbia Glacier (also in Alaska)^8^. Of the three glaciers studied, only Tunabreen experienced sea ice during our study period. The loss of sea ice in spring heralds an increase in calving, but frontal ablation drops to a minimum long before the return of sea ice in January.

### Controls on calving

**Figure 3:**
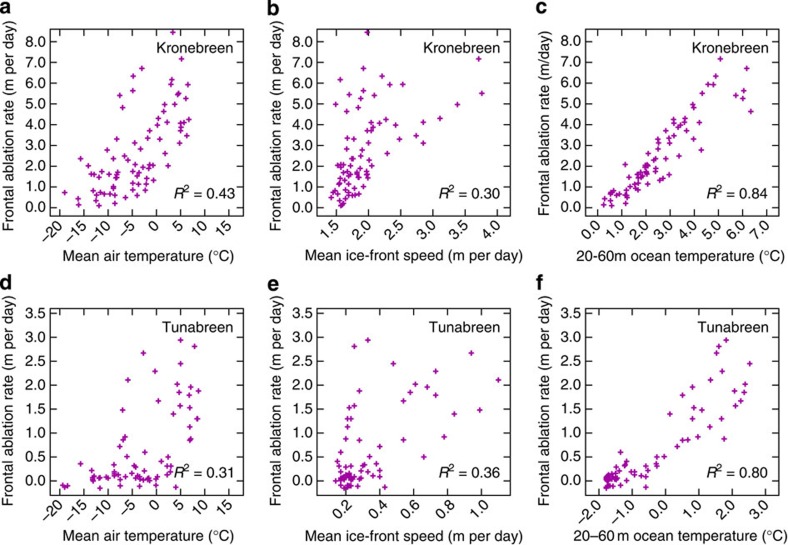
Glacier scatterplots. Correlation data and coefficients of determination for Kronebreen and Tunabreen between frontal ablation rate and: (**a**,**d**) mean air temperature (at Ny Ålesund for Kronebreen and at Longyearbyen for Tunabreen); (**b**,**e**) mean ice-front speed at the glacier terminus; and (**c**,**f**) nearby sub-surface ocean temperature (Kongsfjorden for Kronebreen and Billefjorden for Tunabreen). Note that for Kronebreen in (**b**) frontal ablation rate varies more strongly with ice-front speed when the ice is flowing faster than ∼2.5 m per day.

To explore these interactions in more detail, we further examine relationships between frontal ablation rate and potential controls on calving at Kronebreen and Tunabreen, the glaciers for which we have proximal oceanographic data. Previous studies have investigated calving relationships with water depth, ice thickness and ice speed^8^,^17^, or modelled calving as a process driven by surface melt^18^. Since we need to explain the seasonal variation in frontal ablation, we present relationships with seasonally varying parameters: air temperature, ice speed and sub-surface ocean temperature (Fig. 3). Simple linear regressions between frontal ablation rate and each of the three possible determinants are all statistically significant at the 99% confidence level but demonstrate that sub-surface ocean temperature is by far the strongest predictor of frontal ablation rate at both Kronebreen (*R*^2^=0.84) and Tunabreen (*R*^2^=0.80). Multiple linear regression reveals that including air temperature as a second predictor accounts for only an additional 1.4% of the variance for Kronebreen, and is not significant for Tunabreen. Of the three variables, only sub-surface ocean temperature can adequately explain the seasonal variation in calving behaviour.

## Discussion

Calving events at Kronebreen, Tunabreen and Aavatsmarkbreen, in common with valley-confined tidewater glaciers elsewhere in Svalbard and beyond, typically involve relatively small sections of ice (<50 m across) detaching from the terminus^19^,^20^. Calving events at these glaciers are therefore unlike the massive block rotation events seen in the largest Greenland glaciers, for example, refs 21, 22 or tabular events occurring at ice shelf margins, for example, ref. 23. Nevertheless, these Svalbard glaciers may be typical of medium-sized glaciers terminating in fjords that experience a seasonal incursion of warm water. The submarine melt and calving processes investigated here may therefore be representative of many high-latitude glaciers.

The persistence of frontal ablation at all three glaciers well after winter freeze-up of the glacier surface implies that melt water in crevasses is not a primary control on calving, in contrast to some model findings, for example, ref. 18, and this is further supported by the lack of substantial correlation between local air temperature and frontal ablation. Despite significant contrasts in dynamical behaviour, our studied glaciers have very similar seasonal frontal ablation cycles, implying that dynamic regime also has little influence on the calving process. This is further confirmed by the weak correlation between terminus speed and frontal ablation rate. Surprisingly perhaps, even the initiation of a surge at Aavatsmarkbreen did not seem to influence its frontal ablation rate. These observations imply that frontal ablation rate controls terminus position and is not sensitive to terminus speed (Equation (1)). Whilst a basic consideration of mass balance implies that mean annual calving rates would be expected to correlate well with mean annual ice speed, the weakness of association between ice speed and calving rate on seasonal timescales seen in our data is significant. At Columbia Glacier the seasonal relation between calving rate and ice speed is also much weaker than the annual one^8^. On the other hand, where Kronebreen terminus speeds exceed 2.5 m per day, there is some evidence that frontal ablation rate also varies with glacier speed (Fig. 3b).

The strong correlation between frontal ablation rate and ocean temperature at Kronebreen and Tunabreen is indicative of melt-driven convection at the ice–ocean interface, which is highly sensitive to ambient ocean temperature^24^,^25^. The less strong relationship for Tunabreen may be because our oceanographic data have weaker spatial and temporal relevance. The water temperatures around the depths of the calving fronts of these glaciers are comparable to those elsewhere that give rise to submarine melt on the order of metres per day^1^,^5^,^7^. With the possible exception of the fastest phases of summer flow, this rate of melt appears sufficient to seasonally outpace the delivery of ice to the terminus of our glaciers. Our findings are consistent with melt undercut and collapse being the dominant calving mechanism at these glaciers.

The seasonal cycles of observed ocean temperature in Kongsfjorden and Billefjorden are similar, but the proximity of Kongsfjorden to the WSC and the deep channel that supports a greater exchange of Atlantic-origin waters during summer and autumn^26^ compared with Billefjorden^27^ explain the difference in magnitude of sub-surface temperature between Kongsfjorden and Billefjorden in late September (Fig. 2). The greater frontal ablation rate at Kronebreen, compared with Tunabreen and Aavatsmarkbreen, may therefore be due to the more direct and open connection between this glacier terminus and the source of warm water. In addition, Kronebreen has a much larger ablation area than Tunabreen, and will therefore experience higher summer melt-water discharges. These factors will encourage more efficient subaqueous melting and may help to explain the higher calving rates at Kronebreen^25^,^28^.

On shorter timescales, frontal ablation may be influenced by other factors. For instance, spikes in frontal ablation rates at Kronebreen occasionally coincide with terminus velocity peaks (Fig. 2). It is possible that on these occasions delivery of ice to the glacier terminus outstrips the melt undercut process allowing other calving mechanisms to contribute, and causing the frontal ablation rate to respond more strongly to ice dynamics (Fig. 3b). Alternatively, surface melt-water pulses, as well as promoting faster glacier flow by lubrication, may lead to higher summer melt-water discharge, enhancing convective circulation and melt at the ice front^28^.

Since the relationships between frontal ablation and sub-surface ocean temperature are strong and linear, the data from Kronebreen and Tunabreen (Fig. 3) are in keeping with a simple linear calving law:


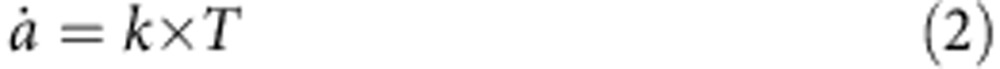


Where 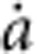 is the frontal ablation rate (m per day), *T* is the temperature at a depth appropriate for each glacier (here 20–60 m) and the coefficient *k* captures the nature of the heat transfer in this setting (1.015 for Kronebreen; 0.35 for Tunabreen). General calving laws of this type, perhaps incorporating other variables such as melt-water discharge, might be developed using data from further glaciers.

We propose that submarine melt is an integral part of a mechanism that promotes calving by undercut and collapse, rather than a process which renders calving potentially unimportant^2^. Where terminus ice velocity is matched or outpaced by submarine melt and undercut calving, a glacier will be stable or will retreat at a rate dependent on the availability of warm water at depth and the circulation of that water through estuarine and glacial discharge processes. Where the ice velocity is greater than the rate at which ice can be removed by melting, the front may be able to advance to a position where other calving processes come into play^29^. This view is consistent with the theoretical framework proposed by Benn *et al*.^30^, in which “first-order” calving processes (determined by the large-scale velocity structure of the glacier) provide the ultimate limit on glacier extent, while “second-order” processes (including melt undercutting) may cause more rapid calving and glacier retreat. The present study, and work elsewhere, for example, ref. 2, indicates that second-order processes currently dominate in some regions. First-order calving may be the rate-limiting process at only the fastest outlet glaciers, for example, ref. 22 or in the coldest ocean settings, for example, ref. 23, these representing only a part of the global ice–ocean interface. Knowledge of submarine melt rates, or much better availability of high-resolution satellite data such as those used in this study, are therefore required to properly understand tidewater calving mechanisms and rates in full.

We have investigated in unprecedented detail the seasonal variation in calving rates at three dynamically contrasting glaciers in Svalbard and their relationships to environmental controls. We find that rates of frontal ablation are not dependent on glacier dynamics, nor reduced by the onset of glacier surface freeze-up, but closely follow the temperature of the fjord waters at depth. Our findings imply that submarine melt undercut and collapse is the dominant calving mechanism at these glaciers, and that frontal ablation is therefore controlled primarily by ocean temperatures. The calving process of melt undercut and collapse may dominate frontal ablation at any glacier where it can match or outpace the delivery of ice to the glacier terminus. This opens up the possibility of being able to predict the response of many tidewater glaciers to oceanographic forcing. We have identified a growing need to assess sub-surface temperatures at tidewater glacier margins and to evaluate the relative prevalence of melt undercut versus other calving processes.

## Methods

### Satellite image data

**Figure 4:**
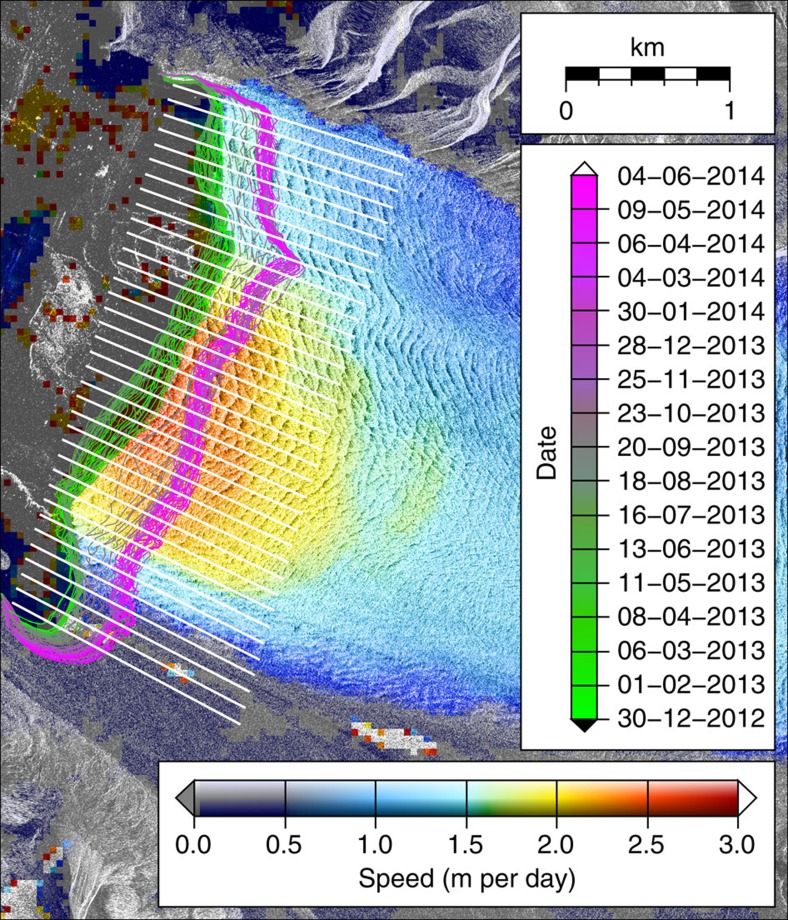
Kronebreen velocity map. Example TerraSAR-X speed map of Kronebreen from January 2013 illustrating glacier surface texture exploited by feature tracking, typical quality of speed data acquired right up to the ice front, and variation in ice-front position over the observation period (green to magenta lines). The ice-front measurement flow-lines along which ice velocity and retreat rate are measured are shown in white.

Feature tracking was applied to TerraSAR-X image pairs in slant range using correlation windows of 200 × 200 pixels spaced every 20 pixels, for example, ref. 31, and subsequently ortho-rectified to a pixel size of 40 m using a Digital Elevation Model. Ice fronts were manually digitized from dB-scaled images ortho-rectified to a pixel size of 2 m using the same Digital Elevation Model. Ice-front position change was derived by locating the intersections between each ice front and a set of flow-lines equally spaced at 100 m intervals across the active glacier terminus width (Fig. 4), and finding the distance between these intersections for each image pair. Surface velocities were extracted at the locations of these ice-front intersections, and the frontal ablation rate at each intersection was calculated using equation (1). These “specific frontal ablation rates” were averaged to produce a mean frontal ablation rate for the entire glacier width for each image pair. This is the first report of frontal ablation calculated using surface velocity measured at the evolving ice front.

Uncertainties in surface velocity *U*_*T*_ are estimated to be <0.4 m per day, and comprise co-registration error (±0.2 pixels) and error arising from unavoidable smoothing of the velocity field over the feature-tracking window size (400 × 400 m). Uncertainties in the rate of ice-front position change d*l*/d*t* are estimated to be <2.0 m per day and are dominated by digitization error (±4 pixels for each of 2 images over 11 days). The combined estimated specific frontal ablation rate error of up to 2.4 m per day is too large to confidently assess the variation in specific frontal ablation rate between individual measurements across the terminus. The RMS frontal ablation rate error for ∼30 flow-line crossing points of ∼0.4 m per day, however, is sufficiently small to allow mean frontal ablation rates to be meaningfully compared between glaciers.

### Weather and sea-surface data

We used daily mean air temperatures and precipitation totals from the Norwegian Meteorological Institute (www.yr.no), measured at the closest available weather station. For Kronebreen and Aavatsmarkbreen this was Ny Ålesund and for Tunabreen, Longyearbyen.

To characterize the fjord surface conditions, we used daily Operational Sea-Surface Temperature and Sea Ice Analysis (OSTIA) data supplied by the Met Office (Crown Copyright 2014). OSTIA SSTs are produced at ∼5 km resolution using an optimal interpolation method to incorporate *in situ* and satellite observations, and sea ice fractional coverages are based on data from the EUMETSAT Ocean & Sea Ice Satellite Application Facility^32^.

We also present data on the presence of fast ice (sea ice frozen to the shoreline) from the Norwegian Ice Service (wms.met.no/icechart). Ice charts are derived by manual classification of satellite images^33^.

### Ocean sub-surface temperature data

Sub-surface fjord water temperatures were measured using moored instrumentation as described by Cottier *et al*.^26^ Temperature sensors, with a precision of better than 0.1 °C after calibration, were positioned from ∼20 m below the surface to within 15 m of the seabed. The moorings typically include 10 sensors over the full 200 m depth with 4–5 sensors at 10 m intervals over the 20–60 m depth range used in this study. To characterize sub-surface ocean temperatures at Kronebreen and Aavatsmarkbreen, we used mooring data from Kongsfjorden (78° 58′ N 11° 48′ E; Fig. 1) available for the entire 2013–2014 study period with the exception of September 2013. For Tunabreen in Tempelfjorden, we used the closest available data from neighbouring Billefjorden (78° 39′ N 16° 41′ E; Fig. 1). These data may be considered a reliable indicator of near-surface oceanographic conditions at Tunabreen because the two fjords are similar in shape and connect adjacently to the same Isfjorden system. The Billefjorden data were available only until September 2013 so our best oceanographic comparison is to the mean value over the period 2008 and 2013.

The mean temperature in the 20–60 m depth range for the two moorings was calculated at weekly intervals to provide an appropriate comparison with the calving time series derived every 11 days or better. The choice of a 60 m maximum for Kongsfjorden was dictated by the depth at which water in the inner fjord is connected to the outer fjord^34^, and for Billefjorden by the presence of a sill at ∼50 m (ref. 22). In addition, both Kronebreen and Tunabreen are grounded in ∼70 m of water.

## Additional information

**How to cite this article:** Luckman, A. *et al*. Calving rates at tidewater glaciers vary strongly with ocean temperature. *Nat. Commun*. 6:8566 doi: 10.1038/ncomms9566 (2015).
